# Metabolic engineering of *Clostridium beijerinckii* to improve glycerol metabolism and furfural tolerance

**DOI:** 10.1186/s13068-019-1388-9

**Published:** 2019-03-09

**Authors:** Chidozie Victor Agu, Victor Ujor, Thaddeus Chukwuemeka Ezeji

**Affiliations:** 1 Department of Animal Sciences and Ohio State Agricultural Research and Development Center (OARDC), The Ohio State University, 305 Gerlaugh Hall, 1680 Madison Avenue, Wooster, OH 44691 USA; 2 INanoBio Inc., 320 Logue Ave., Suite 212, Mountain View, CA 94043 USA; 3 Bioenergy and Biological Waste Management Program, Agricultural Technical Institute, The Ohio State University, 1328 Dover Road, Wooster, OH 44691 USA

**Keywords:** Metabolic engineering, Butanol, Glycerol, Furfural, *C. beijerinckii*, *C. pasteurianum*, Glycerol dehydrogenase, Dihydroxyacetone kinase

## Abstract

**Background:**

Inefficient utilization of glycerol by *Clostridium beijerinckii* (*Cb*) is a major impediment to adopting glycerol metabolism as a strategy for increasing NAD(P)H regeneration, which would in turn, alleviate the toxicity of lignocellulose-derived microbial inhibitory compounds (LDMICs, e.g., furfural), and improve the fermentation of lignocellulosic biomass hydrolysates (LBH) to butanol. To address this problem, we employed a metabolic engineering strategy to enhance glycerol utilization by *Cb*.

**Results:**

By overexpressing two glycerol dehydrogenase (Gldh) genes (*dhaD1* and *gldA1*) from the glycerol hyper-utilizing *Clostridium pasteurianum* (*Cp*) as a fused protein in *Cb*, we achieved approximately 43% increase in glycerol consumption, when compared to the plasmid control. Further, *Cb*_*dhaD1 *+ *gldA1* achieved a 59% increase in growth, while butanol and acetone–butanol–ethanol (ABE) concentrations and productivities increased 14.0%, 17.3%, and 55.6%, respectively, relative to the control. Co-expression of *dhaD1 *+ *gldA1* and *gldA1 *+ dihydroxyacetone kinase (*dhaK*) resulted in significant payoffs in cell growth and ABE production compared to expression of one Gldh. In the presence of 4–6 g/L furfural, increased glycerol consumption by the *dhaD1 *+ *gldA1* strain increased cell growth (> 50%), the rate of furfural detoxification (up to 68%), and ABE production (up to 40%), relative to the plasmid control. Likewise, over-expression of [(*dhaD1 *+ *gldA1*) *dhaK*] improved butanol and ABE production by 70% and 50%, respectively, in the presence of 5 and 6 g/L furfural relative to the plasmid control.

**Conclusions:**

Overexpression of *Cp gldhs* and *dhaK* in *Cb* significantly enhanced glycerol utilization, ABE production, and furfural tolerance by *Cb*. Future research will address the inability of recombinant *Cb* to metabolize glycerol as a sole substrate.

**Electronic supplementary material:**

The online version of this article (10.1186/s13068-019-1388-9) contains supplementary material, which is available to authorized users.

## Background

Due to the finite nature of crude oil, coupled with environmental concerns associated with the consumption of fossil fuels, biofuels have gained significant attention over the past few decades. For instance, biodiesel production in the US has increased substantially from 75 million gallons per annum in 2005 to approximately 2 billion gallons in 2017 [1, 2]. Notably, ~ 10% of the oil feedstock used for biodiesel production ends up as crude glycerol. Consequently, in 2017, an estimated 200 million gallons of crude glycerol was available as a potential fermentation feedstock. Consequently, crude glycerol is a financial and environmental liability due to the attendant cost of disposal [3]. Crude glycerol must be refined to a higher purity to remove contaminants such as alcohol and salts before it can be applied in industrial chemical processes [3]. However, the purification process, which costs ~ $0.2/lb, excluding the cost of transportation to the glycerol refinery increases the retail price of glycerol from $0.05/lb to about $0.60–$0.90/lb, representing a 300% increase [3]. More importantly, glycerol biorefineries and glycerol markets are currently overwhelmed by excess supply of crude and refined glycerol, respectively.

In light of the need to manage this glycerol glut, it is imperative to develop microbial systems for bioconversion of glycerol into value-added products. Fermentation of glycerol by microbes such as *Clostridium*, *Klebsiella*, *Citrobacter*, and *Enterobacter* species has emerged as a desirable channel for disposing of excess crude glycerol. The use of solventogenic *Clostridium* species for glycerol fermentation is attractive, largely because of the cost-effective nature of microbial fermentation [4, 5]. Thus, glycerol has emerged as an alternative non-food fermentation substrate for microbial production of biofuels and other microbe-derived chemicals. Moreover, glycerol catabolism generates two additional moles of reducing equivalents relative to the consumption of molar equivalent of glucose [6–9]. The resulting reducing power from glycerol catabolism has been shown to facilitate detoxification of lignocellulose-derived microbial inhibitory compounds (LDMICs). Another non-food substrate, lignocellulosic biomass (LB)—such as corn stover, switchgrass, *Miscanthus*, sawdust, and wood shavings—is presently abundant. However, due to the recalcitrance of LB to depolymerization, LB feedstocks must first be pretreated to ensure efficient release of fermentable sugars by saccharolytic enzymes during hydrolysis [10, 11]. Notably, LB pretreatment generates LDMICs, especially furaldehydes (e.g., furfural) and phenolic compounds that limit the fermentation of lignocelluloses-derived sugars to fuels and chemicals [12].

In light of these two challenges—glycerol glut and LDMIC-mediated toxicity—we explored metabolic engineering as a tool for channeling the excess reducing equivalents from glycerol catabolism towards in situ furfural detoxification during fermentation by *Cb* [13]. Previously, we had identified increased NAD(P)H regeneration as the underpinning for improvements in the fermentation profile (increased growth and solvent production) of glycerol-supplemented, furfural-challenged *Cb* cultures (although glycerol utilization by *Cb* was considerably low, when compared to glucose consumption) [13]. Increasing glycerol utilization by *Cb*, therefore, represents a rational approach, not only for increasing furfural detoxification (bioabatement) and butanol production by coupling excess NAD(P)H resulting from glycerol catabolism to furfural reduction and butanol biosynthesis, but also as a cost-effective solution to glycerol glut. Different authors have demonstrated that glycerol has to be supplied at a high concentration; ~ 1:2 molar ratio of glucose to glycerol (36 g/L glucose: 36.1 g/L glycerol), to minimize glucose-mediated repression of glycerol utilization (carbon catabolite repression) [13–15]. Consequently, large amounts of glycerol are left unutilized post fermentation, which currently constitutes a major economic and technical drawback to this approach. A previous attempt to adapt *Cb* to glycerol as a sole carbon source was not successful [16]. In fact, no *Cb* strain capable of growth on glycerol as a sole source of carbon was isolated following prolonged incubation or repeated transfer to fresh glycerol-containing medium [16].

We, therefore, hypothesized that wild-type *Cb* harbors significant metabolic roadblocks to glycerol catabolism, which could be circumvented by metabolic rewiring of the glycerol catabolic machinery of *Cb* via metabolic engineering. Towards this goal, we identified *Clostridium pasteurianum* (*Cp*), an efficient hyper-glycerol-utilizing bacterium [17–19] as a donor of efficient glycerol catabolic repertoire. However, a number of salient factors justify retaining *Cb* as the model butanol-producing cell factory. First, although *Cp* can utilize glycerol as a mono-substrate to produce butanol and 1,3-propanediol, it does not efficiently transition to solventogenesis during fermentation of mixed sugar substrates, as evidenced by butyric acid accumulation as the major fermentation product [17–19], hence, limiting the use of *Cp* to ferment LB hydrolysates to butanol. Second, unlike *Cp*, the tolerance of *Cb* to LDMIC has been extensively studied [10, 20–22]. Lastly, *Cb* tolerates butanol toxicity better than *Cp* [16, 18, 23].

**Fig. 1:**
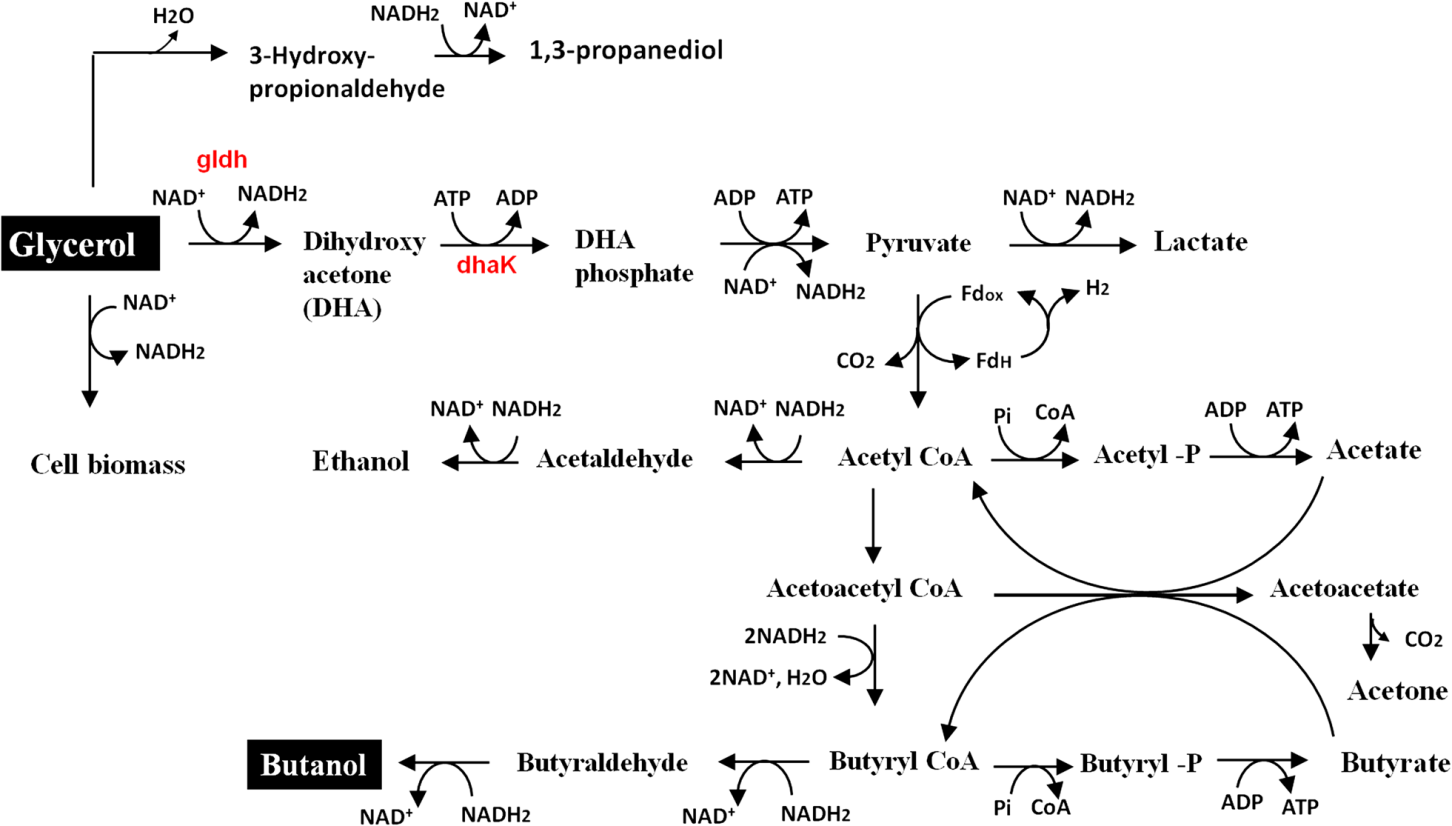
Schematic representation of combined pathways for glycerol utilization in *C. beijerinckii* and *C. pasteurianum*. The pathway is abridged—additional steps between Acetoacetyl CoA and Butryl CoA are not included

Glycerol catabolism to butanol by solventogenic *Clostridium* species proceeds via an initial two-step reaction that feeds the resulting intermediates directly into the glycolytic pathway. First, NAD^+^-dependent glycerol dehydrogenase (Gldh) catalyzes the oxidation of glycerol to dihydroxyacetone (DHA). Next, dihydroxyacetone kinase (DhaK) catalyzes the phosphorylation of DHA to dihydroxyacetone phosphate (DHAP), a glycolytic intermediate (Fig. 1). In this study, we focused on Gldh overexpression in *Cb*, not only because it catalyzes an NAD(P)H-generating reaction, but also because of the multiplicity Gldh in *Cp*. While *Cb* has only one glycerol dehydrogenase gene, *Cp* has four copies (*dhaD1*, *dhaD2*, *gldA1*, and *gldA2*), that vary significantly in terms of protein sequence and secondary structure. Both microorganisms have a single copy of *dhaK*. Therefore, we sought to systematically overexpress these glycerol catabolic genes from *Cp* in *Cb*, thereby constructing *Cb* strains that can efficiently co-metabolize glycerol and lignocellulosic sugars to butanol. Preliminary studies did not reveal marked differences in glycerol utilization and solvent production by different recombinant *Cb* strains expressing the different *gldhs* from *Cp*. Therefore, in the current study, *dhaD1*, *gldA1* and *dhaK* from *Cp* were cloned in *Cb. DhaD2* and *GldA2* will be targeted in future studies. To the best of our knowledge, this is the first attempt at metabolic engineering of *Cb* specifically for enhanced glycerol utilization, as a means of increasing tolerance to LDMICs such as furfural.

## Results

### Protein sequence analyses and secondary structure alignments of *Cp* and *Cb* Gldh and DhaK

Whole genome analysis revealed four copies of *gldh* in *Cp* and one *dhaK*. Conversely, *Cb* has a single copy of each gene. NCBI Blast*p* analysis revealed considerable sequence differences among all four Cp Gldhs, and between *Cp* Gldhs and the single *Cb* Gldh (Additional file 1: Table S1). Similarly, the DhaKs from *Cp* and *Cb* vary significantly in terms of their protein sequences (Additional file 1: Table S3). Alignments of the secondary structures of these proteins revealed significant structural disparities among them, as indicated by the resulting high root mean square deviations (RMSD; Additional file 1: Table S2). Because the alignment of the glycerol dehydrogenases, DhaD1 and GldA1 from *Cp,* produced the least RMSD value, which implies closely matched secondary structures, the genes encoding these two enzymes were, therefore, targeted for initial overexpression as fused proteins with the DhaK gene (from *Cp*) in *Cb*.

### Overexpression of *Cp* GldA1 and DhaD1 as single proteins in *Cb* improved growth with marginal increases in solvent production

To generate recombinant *Cb* strains that can efficiently utilize glycerol as a carbon source, genes that encode Gldhs and DhaK in the hyper-glycerol-utilizing *Cp* were systematically overexpressed in *Cb*. Two of the four genes that encode Gldhs (*gldA1* and *dhaD1*) in *Cp* were first overexpressed as single proteins in *Cb* under the control of an inducible acetoacetate decarboxylase-*adc* (pWUR459) or a constitutive thiolase (pWUR460) promoter to generate four recombinant *Cb* strains: *Cb* pWUR459_*gldA1*, *Cb* pWUR460_*gldA1*, *Cb* pWUR459_*dhaD1*, and *Cb* pWUR460_*dhaD1*. The empty plasmid control (*Cb* pMTL500E) consisted of the backbone plasmid without insert and promoter.

**Fig. 2:**
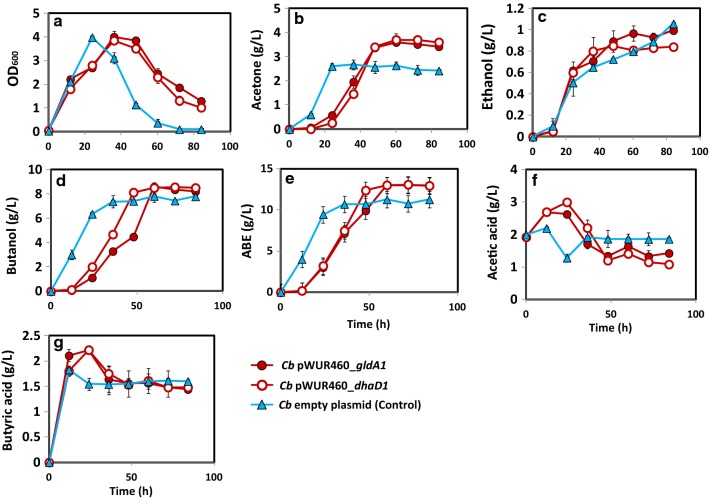
The Fermentation profiles of constitutive *Cb* pWUR460_*dhaD1* and *Cb* pWUR460_*gldA1* during growth in glycerol-supplemented glucose medium. **a** Cell growth in terms of OD_600_, **b** acetone, **c** ethanol, **d** butanol, **e** ABE, **f** acetic acid, **g** butyric acid

**Fig. 3:**
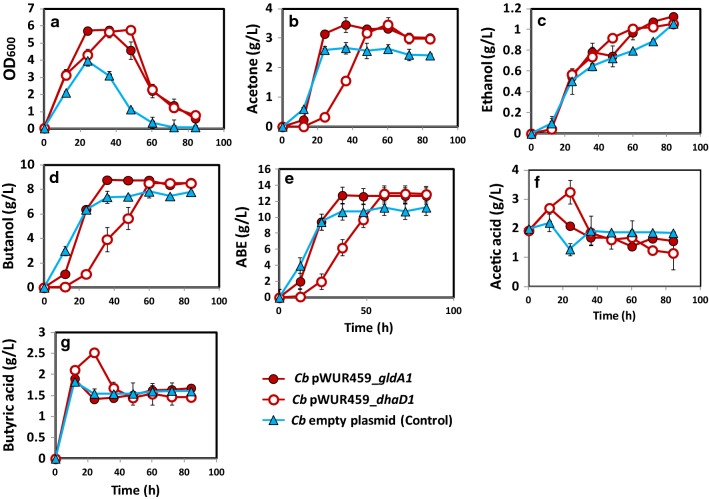
Fermentation profiles of inducible *Cb* pWUR459_*dhaD1* and *Cb* pWUR459_ *gldA1* during growth in glucose + glycerol medium. **a** Cell growth in terms of OD_600_, **b** acetone, **c** ethanol, **d** butanol, **e** ABE, **f** acetic acid, **g** butyric acid

The fermentation profiles of *Cb* pWUR460_*gldA1* and *Cb* pWUR460_*dhaD1* (constitutively expressing *Gldhs*) are shown in Fig. 2, while those of *Cb* pWUR459_*gldA1* and *Cb* pWUR459_*dhaD1* (inducible expression of *Gldhs*) are shown in Fig. 3. With both constitutive and inducible overexpression, we observed similar fermentation profiles in terms of cell growth and solvent production between strains overexpressing *gldA1* and *dhaD1*, indicating similar catalytic activity for both enzymes towards glycerol. Expectedly, the fermentation profiles of recombinant *Cb* strains—*Cb_gldA1* and *Cb*_*dhaD1*—showed improved growth and solvent accumulation, relative to the plasmid control. Specifically, when expressed under the control of the constitutive promoter, *gldA1* and *dhaD1* showed marginal increases in cell growth relative to the plasmid control (Fig. 2a). Conversely, overexpression of *gldA1* and *dhaD1* under the control of an inducible promoter increased cell growth  ~ 45% when compared to the plasmid control (Fig. 3a).

Butanol and total ABE production by the inducible *Cb* strains, *Cb* pWUR459*_gldA1* and *Cb* pWUR459*_dhaD1* were 8.75 g/L and 12.7 g/L, and 8.5 and 12.97 g/L, respectively (Fig. 3d, e). These values were comparable to the constitutive strains—*Cb* pWUR460_*gldA1* produced 8.6 g/L and 13 g/L butanol and ABE, respectively; while, *Cb* pWUR460_*dhaD1* produced 8.56 g/L and 13.01 g/L, respectively (Fig. 2d, e). Overall, butanol and total ABE concentrations were > 15% higher in the recombinant *Cb* strains, *Cb_gldA1* and *Cb_dhaD1* relative to the plasmid control. The ABE productivities of *Cb* pWUR460_*gldA1* and *Cb* pWUR460_*dhaD1* were similar; 0.22 g/L/h, which was 17% higher than that of the empty plasmid control (0.18 g/L/h). Interestingly, despite significant increases in cell growth by the induced *gldA1*- (*Cb* pWUR459_*gldA1*) and *dhaD1*-expressing (*Cb* pWUR459_*dhaD1*) strains relative to the strains bearing constitutively expressed *gldA1* and *dhaD1* and the plasmid control, these increases did not translate into increased total ABE production (Figs. 2a, 3a).

In regards to acid production and re-assimilation, constitutively expressed *gldA1* and *dhaD1* accumulated higher acetic and butyric acid levels at 12 h and 24 h of fermentation, when compared to the plasmid control. Both strains, however, re-assimilated > 51% of the total acetic and butyric acids produced relative to 14.6% acetic acid and 12.02% butyric acid re-assimilation by the plasmid control (Fig. 2f, g). The induced *gldA1* and *dhaD1* showed similar acid production and re-assimilation patterns as the constitutive strains (Fig. 3f, g).

### Overexpression of *Cp* GldA1 (or DhaD1) with DhaK led to greater payoffs compared to overexpression of a single *gldh*

By linking two enzymes of the same metabolic pathway, Dueber et al. [24] observed improvements in the local concentration of enzyme activity, with concomitant increase in the conversion rates of metabolic intermediates. This was the basis for fusing glycerol catabolic enzymes from *Cp* in *Cb*, such that active sites exist in close proximity, whereby the product of one reaction is potentially utilized rapidly by the adjacent enzyme (Additional file 1: Figure S2). To ensure maximum payoff in terms of butanol production, *Cp dhaK* was covalently linked with each of *gldA1* and *dhaD1*, thereby expressing them as fused proteins. Because overexpression of *gldA1* and *dhaD1* as single proteins offered similar benefits, *Cb* was transformed with only the recombinant plasmids carrying *gldA1 *+ *dhaK* to generate *Cb* pWUR459_[*gldA1 *+ *dhaK*] and *Cb* pWUR460_[*gldA1 *+ *dhaK*]. The two strains were used to conduct batch ABE fermentations in glucose + glycerol medium (1:2 molar ratio, respectively) supplemented with 20 µg/mL erythromycin.

**Fig. 4:**
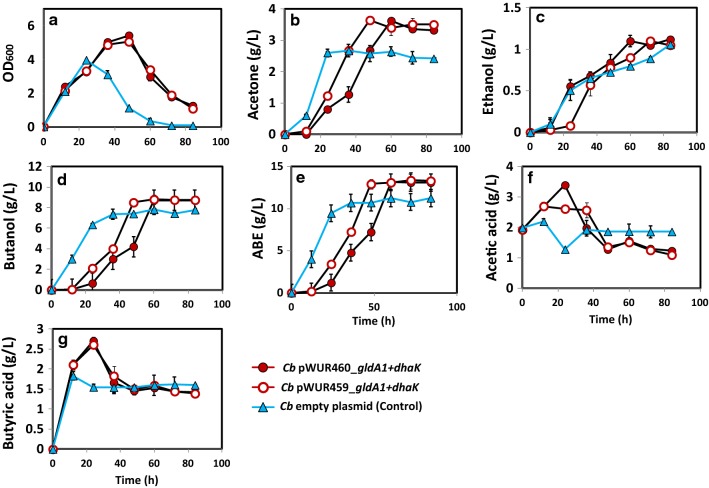
Fermentation profiles of *Cb* pWUR460_*gldA1 *+ *dhaK* and *Cb* pWUR459_*gldA1 *+ *dhaK* during growth on glucose + glycerol medium. **a** Cell growth in terms of OD_600_, **b** acetone, **c** ethanol, **d** butanol, **e** ABE, **f** acetic acid, **g** butyric acid

Figure 4 compares the fermentation profiles of *Cb* pWUR459_[*gldA1 *+ *dhaK*] and *Cb* pWUR460_[*gldA1 *+ *dhaK*] to those of the plasmid control. Cell growth increased significantly (40%) in the cultures of *Cb* pWUR460_[*gldA1 *+ *dhaK*] and *Cb* pWUR459_[*gldA1 *+ *dhaK*], relative to the plasmid control. Similarly, butanol and ABE production increased 11.7% and 17%, respectively, with *Cb* pWUR460_[*gldA1 *+ *dhaK*] and 13% and 14%, respectively, with *Cb* pWUR459_[*gldA1 *+ *dhaK*], relative to the plasmid control. Also, the ABE productivity of *Cb* pWUR460_[*gldA1 *+ *dhaK*] and pWUR459_[*gldA1 *+ *dhaK*] (0.22 g/L/h and 0.36 g/L/h, respectively) increased 22.2% and 50% relative to the plasmid control (0.18 g/L/h; Table 1). Although, cell growth and ABE productivity increased 21.8% and 11.4%, respectively, with inducible expression (*Cb* pWUR459_[*gldA1 *+ *dhaK*]), when compared to constitutive expression (*Cb* pWUR460_[*gldA1 *+ *dhaK*]; Fig. 4; Table 1), final butanol and ABE concentrations were similar for both strains. This result is consistent with the observation for constitutive versus inducible overexpression of GldA1 or DhaD1 as single proteins, indicating that inducible overexpression of these proteins results in accumulation of higher cell biomass, albeit with no significant payoffs in solvent accumulation.

**Table 1:** Substrate utilization, ABE productivity and yield of recombinant *C. beijerinckii* (*Cb*) strains during fermentation of glycerol + glucose medium

Recombinant *Cb* strain	Glycerol utilized		Glucose utilized		ABE		
	Conc. (g/L)	% Improvement vs. control	Conc. (g/L)	Rate (g/L/h)	Conc. (g/L)	*X* (g/L/h)	*Y* (g/g)
pWUR460_*gldA1*	21.61 ± 2^a^	25.2	35.1 ± 2.3	0.96^a^	13 ± 0.01^a^	0.22^a^	0.24^a^
pWUR459_*gldA1*	23.1 ± 2.1^a^	33.8	34.8 ± 4.5	1.24^b^	12.8 ± 1.2^a^	0.35^b^	0.22^a^
pWUR460_*dhaD1*	22 ± 0.12^a^	27.35	35 ± 1.9	1.01^a^	13.1 ± 0.1^a^	0.22^a^	0.23^a^
pWUR459_*dhaD1*	23.4 ± 3.1^a^	35.5	35.1 ± 1.3	1.3^b^	13 ± 0^a^	0.22^a^	0.23^a^
pWUR460_*gldA1 *+ *dhaK*	23.67 ± 0^a^	37.14	35.2 ± 1.2	0.98^a^	13.2 ± 1.1^a^	0.22^a^	0.22^a^
pWUR459_ *gldA1 *+ *dhaK*	24.4 ± 0.8^a^	41.4	35.1 ± 0.8	1.18^c^	12.8 ± 0.7^a^	0.36^b^	0.22^a^
pWUR460_*dhaD1 *+ *gldA1*	24.6 ± 0.1^a^	42.94	35 ± 0.2	1.01^a^	13.2 ± 0.1^a^	0.28^a^	0.22^a^
pWUR459_[(*dhaD1 *+ *gldA1*) *dhaK*]	23.2 ± 0^a^	28.62	34.8 ± 1.2	1.22^a^	12.5 ± 0.5^a^	0.26^a^	0.22^a^
pMTL500E P_*adc*_^(−)^ P_*thl*_^(−)^ (Control)	17.26 ± 1^b^	–	34.8 ± 0.8	1.14^c^	11.3 ± 0.4^b^	0.18^c^	0.22^a^

*X* productivity; *Y* yield

^a,b,c^Fisher’s least significant difference (*p *< 0.1) within parameters for each insert versus the plasmid control. Values with same supesrcript are not significantly different (*p * < 0.1)

Constitutive overexpression of *gldA1* with *dhaK* as a fused protein resulted in significant increase in cell growth (35.8%) when compared to constitutive overexpression of *gldA1* alone. Nevertheless, the difference in butanol production was not profound. Increase in butanol concentration was marginal (1.51%) in favor of *gldA1 *+ *dhaK* when compared to *gldA1* expressed alone (Table 1). ABE concentrations and productivities were similar for both strains (~ 13.2 g/L and 0.22 g/L/h, respectively).

Contrary to constitutive overexpression where a remarkable difference was observed in cell growth between *Cb*_*gldA1* and *Cb*_*gldA1 *+ *dhaK*, inducible overexpression of *gldA1 *+ *dhaK* increased cell growth by only 8%, when compared to *Cb*_*gldA1*. As with the constitutive strains, butanol and ABE concentrations of *Cb* pWUR459_*gldA1 *+ *dhaK* (8.81 g/L and 12.81 g/L, respectively) were similar to those of *Cb* pWUR459_*gldA1* (8.75, and 12.9 g/L, respectively; Figs. 3, 4).

### Co-expression of *Cp gldA1 *+ *dhaD1* as a fused protein improved cell growth relative to overexpression of the single proteins

The basis for linking similar proteins that catalyze the same reaction was to amplify the number of active sites per recombinant protein, thereby potentially increasing the efficiency of substrate conversion. Therefore, *Cp* dhaD1 and gldA1 were covalently tethered with a polyglycine linker, and the gene construct was then cloned into pWUR460 followed by transformation into *Cb*. We sought to ascertain whether co-expression of two Gldhs as a fused protein would lead to more beneficial effects, when compared to the overexpression of a single Gldh. In an effort to further increase glycerol utilization, as observed with *gldA1 *+ *dhaK*—i.e., concomitant overexpression of *Cp* glycerol dehydrogenase and dihydroxyacetone kinase—the fused *dhaD1 *+ *gldA1* was linked to *dhaK* to generate [(*dhaD1 *+ *gldA1*) *dhaK*].

Owing to the potentially large size of the resulting insert [(*dhaD1 *+ *gldA1*) *dhaK*], which may pose some expression problems when overexpressed as single fused protein, *dhaK* in the construct [(*dhaD1 *+ *gldA1*) *dhaK*] was designed to be expressed alone as a single protein, while *dhaD1 *+ *gldA1* was expressed as a fused protein. To avoid likely metabolic burden potentially induced by the large size of the resulting recombinant plasmid, overexpression of [(*dhaD1 *+ *gldA1*) *dhaK*] insert was placed under the control of inducible acetoacetate decarboxylase (*adc*) promoter, in pWUR459. The newly generated *Cb* strains—*Cb* pWUR460_[*dhaD1 *+ *gldA1*] and *Cb* pWUR459_[(*dhaD1 *+ *gldA1*) *dhaK*]—were used to conduct batch ABE fermentation in glucose + glycerol medium (1:2 molar ratio).

**Fig. 5:**
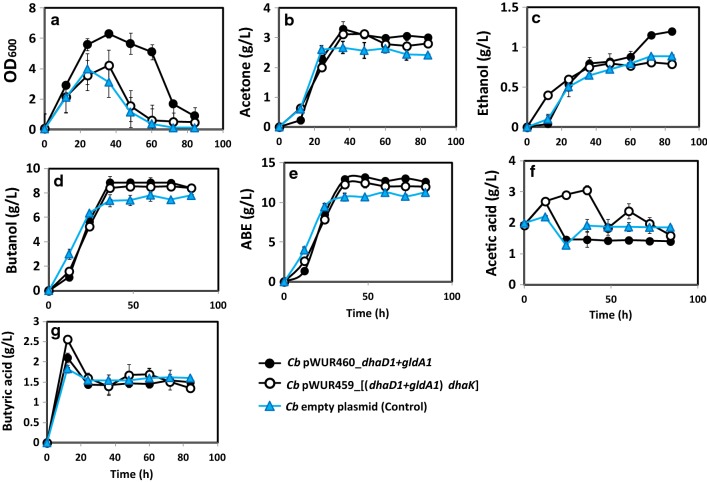
Fermentation profiles of *Cb* pWUR460_*dhaD1 *+ *gldA1* and *Cb* pWUR459_[(*dhaD1 *+ *gldA1*) *dhaK*] during growth on glycerol-supplemented glucose medium. **a** Cell growth in terms of OD_600_, **b** acetone, **c** ethanol, **d** butanol, **e** ABE, **f** acetic acid, **g** butyric acid

Figure 5 shows the fermentation profiles of *Cb* pWUR460_*dhaD1 *+ *gldA1* and *Cb* pWUR459_[(*dhaD1 *+ *gldA1*) *dhaK*] relative to the plasmid control, without furfural supplementation. The fermentation profiles of *Cb* pWUR460_*dhaD1 *+ *gldA1*, pWUR460_*gldA1*, pWUR460_*dhaD1*, and pWUR460_*gldA1 *+ *dhaK* were compared. Cell growth, butanol, and ABE concentrations increased 59%, 14%, and 17.3%, respectively, in *Cb* pWUR460_*dhaD1 *+ *gldA1* relative to the plasmid control (Fig. 5). Likewise, the ABE productivity of *Cb* pWUR460_*dhaD1 *+ *gldA1* was significantly higher (55.6%) when compared to the plasmid control (Table 1). Further, cell growth increased 64% and 57.5% in *Cb* pWUR460_*dhaD1 *+ *gldA1* when compared to pWUR460_*dhaD1* and pWUR460_*gldA1*, respectively. Similarly, butanol concentration and ABE productivity of *Cb* pWUR460_*dhaD1 *+ *gldA1* increased ~ 4% and 27.3%, respectively, relative to *Cb* pWUR460_*dhaD1* and pWUR460_*gldA1* (Table 1).

*Cb* pWUR460_*dhaD1 *+ *gldA1* exhibited 16% higher cell OD_600_ when compared to *Cb* pWUR460_*gldA1 *+ *dhaK* (Figs. 4a, 5a). Likewise, the ABE productivity of *Cb* pWUR460_*dhaD1 *+ *gldA1* was 27% higher than that of *Cb* pWUR460_*gldA1 *+ *dhaK* (Table 1), although the corresponding final butanol and ABE concentrations (8.85 g/L and 13.2 g/L) were similar to those of pWUR460_*gldA1 *+ *dhaK* (8.72 g/L and 13.2 g/L).

Inducible overexpression of fused [*dhaD1 *+ *gldA1*] with *dhaK* as in *Cb* pWUR459_[(*dhaD1 *+ *gldA1*) *dhaK*] increased butanol and total ABE concentrations, and ABE productivity to 8.83%, 12.71%, and 44.4% (*p *< 0.1), relative to the plasmid control (Table 1 and Fig. 5d, e). Although constitutive overexpression of *gldA1* with *dhaK* as a fused protein resulted in significant improvement in cell growth relative to constitutive overexpression of *gldA1* alone, there was no similar improvement in cell growth when [*dhaD1 *+ *gldA1*] was overexpressed with *dhaK*, despite the use of an inducible promoter which was earlier observed to result in higher cell growth (Figs. 2a, 3a; perhaps, this was because *dhaK* was overexpressed as a single protein in [(*dhaD1* +* gldA1*) *dhaK*]. Nonetheless, butanol and ABE concentrations of pWUR459_[*dhaD1 *+ *gldA1*] + *dhaK* (8.52 g/L and 12.7 g/L) were comparable to those of pWUR460_*dhaD1 *+ *gldA1* (8.86 g/L and 13.18 g/L), and significantly higher (*p *< 0.1) than the empty plasmid control (7.81 g/L and 11.25 g/L; Table 1 and Fig. 5d, e).

### Glucose and glycerol consumption by recombinant *Cb* strains

Overall, we observed increase in glycerol consumption by recombinant strains of *Cb-*overexpressing *Cp gldh* and *dhaK* genes relative to the plasmid control (Table 1). Glycerol consumption by *Cb* pWUR460_*gldA1* and *Cb* pWUR460_*dhaD1* increased 25% and 28%, respectively, when compared to the plasmid control (Table 1). Further improvement in glycerol utilization was observed with *Cb* pWUR460_*gldA1 *+ *dhaK,* which utilized 37.1% more glycerol than the empty plasmid control. This represents ~ 10% increase in glycerol consumption when compared to overexpression of Gldh only.

Constitutive overexpression of two Gldh [*dhaD1 *+ *gldA1*] as a fused protein increased glycerol utilization by 43% relative to the plasmid control, thus, representing ~ 14% increase in glycerol consumption relative to overexpression of GldA1 or DhaD1 alone (Table 1). With [(*dhaD1 *+ *gldA1*) *dhaK*], 28.6% increase in glycerol utilization was observed (compared to 43% by *dhaD1 *+ *gldA1*), relative to the plasmid control. Although 28.6% increase is a significant improvement relative to the plasmid control, this increase was still 14% short of the improvement observed with *dhaD1 *+ *gldA1*.

Although the recombinant strains preferred glucose to glycerol as substrate, as evidenced by complete utilization of glucose after 48 h, we found that the rate of glucose utilization by the plasmid control after 12 h of fermentation was slightly higher than those of the constitutively expressed (pWUR460) recombinant *Cb* strains (Table 1). For example, the rate of glucose consumption by the plasmid control was 1.2 g/L/h, whereas the rates of glucose consumption were 0.96, 1.0, 0.98, and 1.0 g/L/h for *Cb* pWUR460_*gldA1*, *dhaD1*, [*gldA1 *+ *dhaK*], and [*dhaD1 *+ *gldA1*], respectively (Table 1). To ascertain whether decreases in the rates of glucose utilization were due to constitutive overexpression of the Gldhs, we determined glucose consumption rates for the inducible overexpression strains. Our results showed that overexpression of Gldhs using the inducible promoter increased the rate of glucose utilization relative to constitutive overexpression and the plasmid control. For example, the rates of glucose consumption by *Cb* pWUR459_*gldA1* and *Cb* pWUR459_ *dhaD1* increased 29.2% and 8.8%, and 29% and 14%, relative to their respective constitutive strains and the plasmid control, respectively (Table 1). Also, *Cb* pWUR459_[*dhaD1 *+ *gldA1*] + *dhaK* showed 7% increase in the rate of glucose consumption when compared to the plasmid control (Table 1). The rate of glycerol consumption by the constitutive *Cb* pWUR460_*gldA1* after 24 h (0.42 g/L/h) significantly increased (16.2%) when compared to the inducible *Cb* pWUR459_*gldA1* (0.37 g/L/h), which in turn was 9% higher than plasmid control (0.34 g/L/h).

### The activities of glycerol dehydrogenases and dihydroxyacetone kinase in the recombinant *Cb* strains

**Fig. 6:**
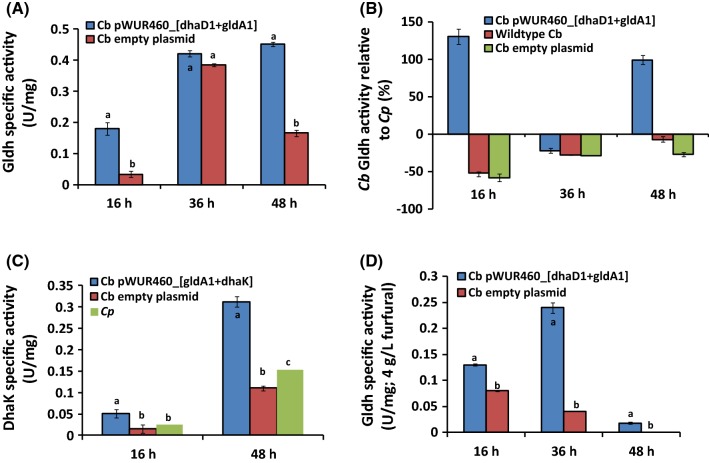
The activities of Gldh and DhaK in cell-free extracts of recombinant *Cb* at 16, 36, and 48 h of growth. **A** Gldh specific activities, **B** Gldh % relative activities compared to wild-type *Cp* Gldh, **C** DhaK specific activities, **D** Gldh activity of *Cb* pWUR460_*dhaD1 *+ *gldA1* challenged with 4 g/L furfural. Different letters (e.g., a, b or c) indicate significance (*p *< 0.1) within each time point

The Gldh and DhaK activities of the recombinant *Cb* strains were measured in crude cell extracts using cells grown in glucose + glycerol medium. Figure 6A shows the Gldh activity of *Cb* pWUR460_*dhaD1 *+ *gldA1* and the empty plasmid control, *Cb* pMTL500E, while Fig. 6B compares their relative activities to *Cp* Gldh. Overall, the Gldh activity increased significantly in the recombinant *Cb* strains when compared to the empty plasmid control (Fig. 6A).

At 16 and 48 h of fermentation, Gldh activity of the plasmid control was 51.3% and 56.7% lower, respectively, when compared to *Cp* Gldh activity (Fig. 6B). However, overexpression of *dhaD1 *+ *gldA1* from *Cp* in *Cb* significantly increased Gldh specific activity by 130.8% and 99.6% at 16 and 48 h, respectively, relative to wild-type *Cp* (Fig. 6B). The specific activities of Gldh at 48 h for *Cb* pWUR460_*gldA1* and *Cb* pWUR460_*dhaD1* (0.34 U/mg and 0.3 U/mg) increased twofold when compared to the plasmid control (0.17 U/mg). At 16 h, the specific Gldh activity of *Cb* pWUR460_*dhaD1 *+ *gldA1* was 4.5- and 3.7-fold higher than that of the empty plasmid control (Fig. 6A). In addition, the Gldh activity of the strain overexpressing *dhaD1 *+ *gldA1* increased 1.5-fold when compared to overexpression of *dhaD1* (or *gldA1*) alone (data not shown).

DhaK specific activity was measured using crude cell extracts of *Cb* pWUR460_*gldA1 *+ *dhaK*. At 48 h, the DhaK activity was 0.31 U/mg, which was significantly higher (2.4-fold) than that of the plasmid control (0.11 U/mg; Fig. 6C). Similarly, the DhaK activity of recombinant *Cb* pWUR460_*gldA1 *+ *dhaK* also increased 2.1-fold relative to the wild-type *Cb* (0.31 U/mg versus 0.1 U/mg, respectively).

### Furfural tolerance of the recombinant *Cb* strains

We previously studied the beneficial role of glycerol supplementation as a strategy for improving furfural detoxification by *C. beijerinckii* [13]. In the current experiment, we sought to exploit improved glycerol utilization accruing from overexpression of *Cp gldh* and *dhaK* in *Cb* as a means to further enhance furfural detoxification. Previously, we had observed that simultaneous supplementation of furfural and erythromycin to the fermentation medium severely inhibits the growth of recombinant *Cb* strains. Therefore, the furfural detoxification experiments were conducted with 15 µg/mL erythromycin. Comparative recombinant Gldh activity was measured at mid-stationary phase (48 h) in *Cb* pWUR460_*dhaD1 *+ *gldA1* and the plasmid control to ascertain recombinant plasmid retention at low erythromycin concentration (15 µg/mL). Indeed, the Gldh activity of *Cb* pWUR460_*dhaD1 *+ *gldA1* in the fermentation medium was significantly higher (2.5-fold) than that of the plasmid control.

Since *Cb* pWUR460_*dhaD1 *+ *gldA1* produced a better fermentation profile than *Cb* pWUR460_*gldA1* (or *dhaD1*) and *Cb* pWUR460_*gldA1 *+ *dhaK*, this strain was selected for fermentation of furfural-challenged glucose + glycerol medium. The use of a constitutive promoter to drive gene expression as in *Cb* pWUR460_*dhaD1 *+ *gldA1* was intended to ensure glycerol utilization from the onset of cell growth, thereby potentially increasing the availability of reducing equivalents crucial to furfural detoxification and butanol formation.

**Fig. 7:**
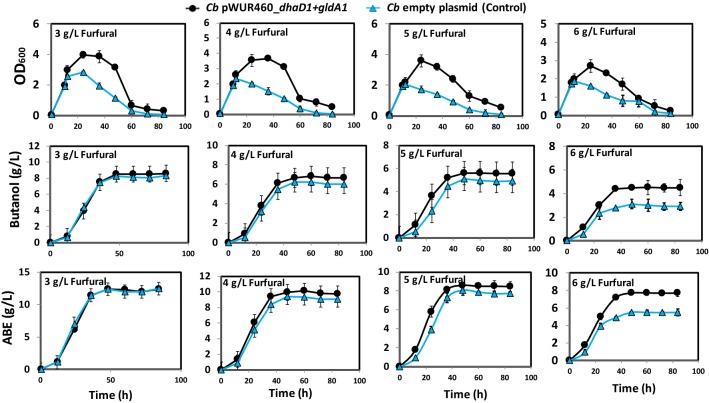
Fermentation profiles of *Cb* pWUR460_*dhaD1 *+ *gldA1* during growth on glycerol-supplemented glucose medium challenged with 3, 4, 5, or 6 g/L furfural. Panel 1: Growth profiles at 3, 4, 5, 6 g/L furfural treatment. Panel 2: Butanol concentrations at 3, 4, 5, 6 g/L furfural treatment. Panel 3: Total ABE concentrations at 3, 4, 5, 6 g/L furfural treatment

Figure 7 and Table 2 show cell growth, butanol and ABE production by furfural (2, 3, 4, 5, and 6 g/L)-challenged *Cb* pWUR460_*dhaD1 *+ *gldA1*. Cell growth increased 55.7, 40.8, 49.2, 45.9, and 30.4% with 2, 3, 4, 5 and 6 g/L furfural treatments, respectively, when compared to the plasmid control. Similarly, butanol concentrations increased 3.4, 10.3, 11.8, and 45.5% (*p *< 0.1) in 3, 4, 5, and 6 g/L furfural-challenged cultures, respectively, when compared to the empty plasmid control (Fig. 7 and Table 2). Likewise, ABE concentration increased 7.6, 6.3, and 40.2% with 4, 5, and 6 g/L furfural challenge, respectively, relative to the control (Fig. 7 and Table 2). In fact, the presence of 2, 3, and 4 g/L furfural increased glycerol consumption by both *Cb* pWUR460_*dhaD1 *+ *gldA1* and the plasmid control, when compared to cultures not supplemented with furfural (data not shown). With 3 and 4 g/L furfural challenge, ~ 20 g/L glycerol was utilized by the plasmid control; whereas, *Cb* pWUR460_*dhaD1 *+ *gldA1* consumed a total of~ 30 g/L glycerol, representing 45% increase in glycerol consumption (Table 2). Similarly, glycerol consumption increased 63% and 77% with 5 and 6 g/L furfural treatments, respectively, relative to the plasmid control. With 6 g/L furfural, the ABE productivity of *Cb* pWUR460_*dhaD1 *+ *gldA1* increased 40% relative to the plasmid control (Table 2).

**Table 2:** Substrate utilization, furfural detoxification, cell growth (optical density; OD_600_), butanol and ABE concentrations (*C*), ABE yield (*Y*) and productivity (*Z*) profiles of *C. beijerinckii* (*Cb*) pWUR460_*dhaD1 *+ *gldA1* grown in furfural (2 to 6 g/L)-supplemented glucose + glycerol medium

Furfural (g/L)	*Cb* strain	Max. cell OD_600_	Butanol			ABE			Substrate utilized		*R* (g/L/h)
			*C* (g/L)	*Y* (g/g)	*Z* (g/L/h)	*C* (g/L)	*Y* (g/g)	*Z* (g/L/h)	Glucose (g/L)	Glycerol (g/L)	
2	pWUR460_ *dhaD1 *+ *gldA1*	5.5 ± 0.1^a^	8.7^a^	0.14^a^	0.18^a^	12.4 ± 0.1^a^	0.20^a^	0.26^a^	35.6 ± 0.1^a^	29.1 ± 1.1^a^	1.36^a^
	Plasmid control	3.5 ± 0.02^b^	8.7 ± 0.1^b^	0.14^a^	0.18^a^	12.1 ± 1.1^a^	0.20^a^	0.20^b^	35.8 ± 1.1^a^	28.9 ± 0.9^a^	1.37^a^
3	pWUR460_ *dhaD1 *+ *gldA1*	4.0 ± 0.03^a^	8.5 ± 0.9^a^	0.13^a^	0.18^a^	12.4 ± 0.5^a^	0.20^a^	0.26^a^	35.8 ± 0.2^a^	29.1 ± 0.1^a^	1.36^a^
	Plasmid control	2.8 ± 0.1^b^	8.2 ± 1.1^b^	0.15^b^	0.17^a^	12.4 ± 0.1^a^	0.19^a^	0.20^b^	36 ± 0.23^a^	20.1 ± 0.9^b^	1.4^a^
4	pWUR460_ *dhaD1 *+ *gldA1*	3.7 ± 0.2^a^	6.9 ± 0.2^a^	0.11^a^	0.14^a^	10.1 ± 0.5^a^	0.16^a^	0.21^a^	35.4 ± 0.5^a^	26.2 ± 1.1^a^	1.15^a^
	Plasmid control	2.4 ± 0.2^b^	6.2 ± 0.4^b^	0.11^a^	0.13^a^	9.4 ± 0.34^b^	0.17^a^	0.20^a^	35.7 ± 0.1^a^	18.6 ± 0.2^b^	1.13^a^
5	pWUR460_ *dhaD1 *+ *gldA1*	3.6 ± 0.2^a^	5.6^a^	0.11^a^	0.12^a^	8.6 ± 0.7^a^	0.17^a^	0.18^a^	25.4 ± 0.2^a^	23.9 ± 0.5^a^	1.38^a^
	Plasmid control	2.1 ± 0.5^b^	5.1 ± 0.8^b^	0.12^a^	0.11^a^	8.1 ± 0.01^b^	0.18^a^	0.17^a^	31.1 ± 0.1^b^	13.1 ± 0.1^b^	0.82^b^
6	pWUR460_ *dhaD1 *+ *gldA1*	2.7 ± 0.9^a^	4.5 ± 0.9^a^	0.2^a^	0.09^a^	7.7^a^	0.34^a^	0.16^a^	9.8 ± 0^a^	13 ± 0.8^a^	1.24^a^
	Plasmid control	1.9^b^	3.1 ± 0.3^b^	0.16^a^	0.06^b^	5.5^b^	0.28^b^	0.12^b^	12 ± 0.1^b^	7.5 ± 0.23^b^	0.96^b^

*C* concentration, *Y* yield, *X* productivity, *R* rate of furfural detoxification

^a,b^Fisher’s LSD (*p *< 0.1) between means of *Cb* pWUR460_ *dhaD1 *+ *gldA1* vs. the plasmid control for each furfural concentration within each parameter

The rates of furfural detoxification by *Cb* pWUR460_*dhaD1 *+ *gldA1* and the plasmid control were similar with 2, 3 and 4 g/L furfural challenge (1.36 and 1.37 g/L/h, respectively, with 2 g/L furfural treatment, 1.36 and 1.4 g/L/h, respectively, with 3 g/L furfural treatment, and 1.15 and 1.13 g/L/h, respectively, with 4 g/L furfural treatment). However, with 5 and 6 g/L furfural treatments, the rates of furfural detoxification by *Cb* pWUR460_*dhaD1 *+ *gldA1* were 1.38 g/L/h and 1.24 g/L/h, respectively; while the rates for the plasmid control were 0.82 g/L/h and 0.96 g/L/h, respectively (Table 2). These represent remarkable increases (67.9% and 29.2%) in the rates of furfural detoxification with 5 g/L and 6 g/L furfural, respectively, by *Cb* pWUR460_*dhaD1 *+ *gldA1*.

The role of glycerol supplementation in facilitating furfural detoxification was evident when the detoxification rates were compared with that of furfural-challenged glucose-only medium. Specifically, furfural detoxification rates were lower for glucose-only medium when compared to glycerol + glucose medium. For example, the rates of furfural detoxification by *Cb* pWUR460_*dhaD1 *+ *gldA1* in glucose-only medium supplemented with 4, 5, and 6 g/L furfural were 0.77, 0.87, and 0.96 g/L/h, respectively. Comparing these rates with 1.15, 1.38 and 1.24 g/L/h obtained with glycerol-supplemented P2 medium challenged with 4, 5 and 6 g/L furfural shows 49.4, 58.6, and 29.2% improvements, respectively, in the rates of furfural detoxification.

Gldh activity of *Cb* pWUR460_*dhaD1 *+ *gldA1* was determined during fermentation of 4 g/L furfural-challenged glucose + glycerol medium (1:2 molar ratio; Fig. 6D). At 16 and 36 h of growth, Gldh activity of *Cb* pWUR460_*dhaD1 *+ *gldA1* increased ~ twofold relative to the empty plasmid control. Interestingly, at 48 h of growth, the empty plasmid control exhibited undetectable Gldh activity, while *Cb* pWUR460_*dhaD1 *+ *gldA1* showed Gldh activity of 0.018 U/mg, thereby implicating overexpression of the recombinant protein in the payoffs observed with glycerol-supplemented furfural-challenged fermentations by *Cb* pWUR460_*dhaD1 *+ *gldA1*.

**Fig. 8:**
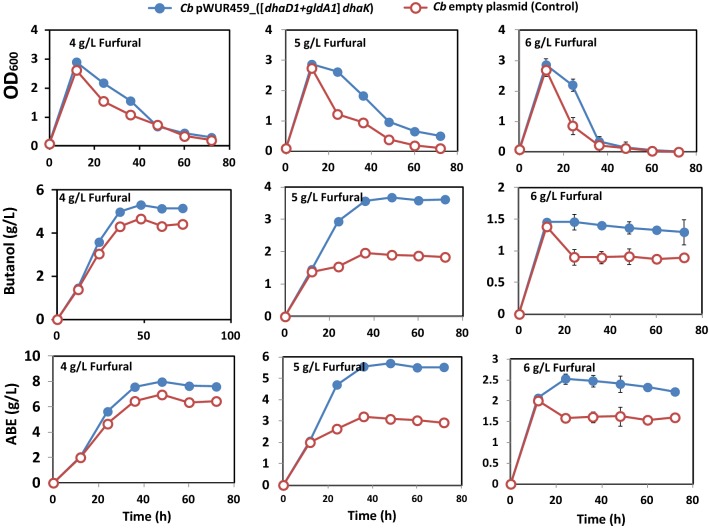
Fermentation profiles of *Cb* pWUR459_[(*dhaD1 *+ *gldA1*) *dhaK*] during growth on glycerol-supplemented glucose medium challenged with 4, 5, or 6 g/L furfural. Panel 1: OD_600_ at 4, 5, 6 g/L furfural treatment. Panel 2: Butanol concentrations at 4, 5, 6 g/L furfural treatment. Panel 3: Total ABE concentrations at 4, 5, 6 g/L furfural treatment

*Cb* pWUR459_[(*dhaD1 *+ *gldA1*) *dhaK*] was also used to ferment glucose + glycerol medium supplemented with 15 µg/mL erythromycin at 0 h, and then challenged with 3, 4, 5, or 6 g/L furfural at 12 h of fermentation. Because inducible *adc* promoter from *C. acetobutylicum* triggers recombinant protein overexpression at early solventogenic phase when sufficient cell biomass has accumulated (18 h; [25]), pWUR459 plasmid was chosen for the overexpression of the large recombinant proteins [(DhaD1 + GldA1) DhaK]. The rationale was to minimize the combined metabolic burden of plasmid replication and furfural toxicity by first accumulating sufficient cell biomass prior to the inception of recombinant protein overexpression and furfural challenge. Notably, *adc* promoter is induced in solventogenic *Clostridium* species at late exponential growth phase during which remarkable amounts of acetic and butyric acid are produced and accumulated by the microorganisms, thus facilitating the switch from acidogenesis to solventogenesis, causing shift from acid accumulation to acid assimilation for solvent biosynthesis [26, 27]. As a result, the *adc* promoter does not require additional exogenous inducer to be turned on, and it is largely functional during the solventogenic growth phase. Figure 8 shows the fermentation profile of *Cb* pWUR459_[(*dhaD1 *+ *gldA1*) *dhaK*] in the presence of 4, 5, and 6 g/L furfural. At 24 h of growth, cell OD was 1.4-, 2.1-, and 2.6-folds at 4, 5, and 6 g/L furfural challenge, respectively, when compared to the plasmid control. With 4 g/L furfural, butanol and ABE concentrations increased 13.9% and 14.5%, relative to the plasmid control. Similarly, with 5 and 6 g/L furfural, butanol and ABE concentrations increased 86.8% and 78.1%, and 53.8% and 52.2%, respectively, when compared to the plasmid control (Fig. 8).

## Discussion

This study was designed to achieve the following objectives: (i) to metabolically engineer *Cb* strains through systematic overexpression of *Cp gldh* and *dhaK* to generate glycerol-utilizing *Cb* strains, (ii) to characterize the phenotypes of the resulting recombinant *Cb* strains in terms of cell growth, ABE production, and glycerol utilization, and (iii) to ascertain whether increased consumption of glycerol translates into increased rate of furfural detoxification and ABE production. To achieve the above objectives, we first overexpressed *Cp gldA1* and *dhaD1* as single proteins in *Cb*. Although there was no difference in the fermentation profiles of both strains, overexpression of each gene in *Cb* enhanced glycerol utilization relative to the plasmid control (25%). This, therefore, suggests that *Cp* glycerol dehydrogenases have specifically evolved to favor glycerol catabolism relative to the single native glycerol dehydrogenase in *Cb*. Whereas this contributes, at least in part, to the superior glycerol utilization by *Cp*, when compared to other *Clostridium* species, the overall glycerol utilization capacity of the resulting recombinant *Cb* strains—which consumed considerably less glycerol than *Cp*—suggests that additional factors beyond individual expression of glycerol dehydrogenases in *Cp* contribute to glycerol utilization by this microorganism (*Cp*).

To further improve glycerol utilization, therefore, we overexpressed *gldA1* with DhaK as a fused protein and observed a 37.1% increase in glycerol utilization when compared to the plasmid control (a further ~ 10% increase when compared to *gldA1* alone). Clearly, the underpinning for this additional payoff accrued from the recombinant DhaK activity. Simultaneous expression of a Gldh and a DhaK from *Cp* in *Cb* was expected to significantly increase flux through the glycerol metabolic shunt, which feeds into the glycolytic pathway, thereby improving growth and ABE production. However, whereas co-expression of Gldh and DhaK from *Cp* increased growth, ABE production and glycerol utilization in *Cb*, when compared to the plasmid control, this did not lead to significant increases relative to co-expression of two Gldhs from *Cp* (DhaD1 + GldA1) in *Cb*. In fact, by overexpressing the two Gldh genes (*dhaD1* and *gldA1*) as a fused protein, we further increased glycerol utilization (to a maximum of 43%), relative to the plasmid control and *gldA1* alone, although cell growth, ABE production and most crucially, glycerol consumption were considerably similar among recombinant *Cb* strains expressing two fused Gldhs (DhaD1 + GldA1) and those expressing a Gldh and a DhaK (GldA1 + DhaK).

Clearly, by combining the catalytic machinery of two Gldhs or a Gldh and a DhaK from *Cp*, we further increased glycerol utilization by *Cb*, as opposed to expressing one Gldh from *Cp* in *Cb*. In light of this, it does appear that the multiplicity of glycerol catabolic genes in *Cp*, particularly Gldhs may account, in part for its superior glycerol metabolism relative to other solventogenic *Clostridium* species. However, it is not clear if all or some of the multiple Gldhs in *Cp* are simultaneously expressed. It would be instructive, therefore, to characterize the expression profiles of Gldhs in *Cp*. Interestingly, co-expression of *dhaK* with *dhaD1 *+ *gldA1* did not increase glycerol utilization any further. The observation that the native DhaK activity in *C. butyricum* is inducible [4, 28] indicates that *dhaK* overexpression in *Cb* may not be critical to drive metabolic flux through the glycerol catabolic shunt, and subsequently towards butanol biosynthesis. More importantly, this suggests that the step catalyzed by Gldh in glycerol metabolism might be the rate-limiting step in glycerol catabolism. This, perhaps explains why *Cp*, a potent utilizer of glycerol, evolved multiple copies of *gldh* genes to promote glycerol catabolism, while harboring a single copy of *dhak* as in *Cb*. Notably, co-expression of *dhaK* with *dhaD1 *+ *gldA1* significantly reduced cell growth when compared to expression of Gldhs alone, or two Gldhs simultaneously, or Gldh and DhaK as a fused protein, although ABE production was not affected (in the strain expressing *dhaK* with *dhaD1 *+ *gldA1*). Perhaps, the large size of the construct [(*dhaD1 *+ *gldA1*) *dhaK*] expressed in this strain (10,745 bp) may have exerted a degree of metabolic burden on this strain, leading to reduced growth. Notably, glycerol utilization and ABE production were not affected in this strain (*Cb*_[(*dhaD1 *+ *gldA1*) *dhaK*]). This indicates that expression of the respective glycerol catabolic genes in this strain, hence flux via the glycerol metabolic shunt, and glycolysis downstream and subsequently towards the ABE pathway were unaffected. Perhaps, metabolic modulations elsewhere, stemming from the expression of a plasmid-borne, large-sized construct impaired growth in *Cb*_[(*dhaD1 *+ *gldA1*) *dhaK*].

To investigate whether enhanced glycerol utilization due to overexpression of *gldhs* would promote furfural tolerance in *Cb*, the recombinant strains were exposed to furfural. Studies have shown that the toxic effects of furfural on microbial physiology include inhibition of specific enzymes and NAD(P)H depletion [11, 22, 29]. In light of this, we measured the activity of NAD(P)H-generating Gldh in crude cell extracts of *Cb* grown in furfural-supplemented glycerol + glucose medium. Interestingly, the Gldh activity of *Cb*_*dhaD1 *+ *gldA1* exposed to 4 g/L furfural was remarkably higher than that of the plasmid control (Fig. 6D). Based on this, we conclude that the significant payoffs in furfural tolerance in the recombinant *Cb* strains relative to the control were due to overexpression of glycerol catabolic genes (*dhaD1 *+ *gldA1* and [(*dhaD1 *+ *gldA1*) *dhaK*]), leading to enhanced glycerol consumption, and consequently, excess reducing equivalents for furfural detoxification and butanol production (Additional file 1: Figure S4, Table 2). In fact, recombinant *Cb* strains demonstrated more rapid rates of furfural reduction to the less toxic furfuryl alcohol, especially with increasing furfural concentration. For instance, whereas *Cb* pWUR460_*dhaD1 *+ *gldA1* and the empty plasmid control showed similar rates of furfural reduction with 2 and 3 g/L furfural, the rates of furfural reduction by *Cb* pWUR460_*dhaD1 *+ *gldA1* were 67.9% and 29.2% greater than those of the empty plasmid with 5 and 6 g/L furfural, respectively (Additional file 1: Figure S4).

## Conclusions

Our results demonstrate that overexpression of *Cp dhaD1* and *gldA1* either as single or a fused protein or in combination with *dhaK* enhanced glycerol consumption and improved furfural tolerance by the recombinant *Cb* strains. Despite improved glycerol consumption by the recombinant *Cb* strains during growth in a glucose + glycerol medium, there were no marked increases in cell optical density during the growth of these strains in a glycerol-only medium (data not shown). Clearly, the inability of *Cb* to metabolize glycerol as a sole substrate stems from other factors beyond the lack of superior glycerol catabolic enzymes (such as those found in *Cp*). One of such factors is the likely absence of a short excess NAD(P)H-dissipation pathway during oxidation of glycerol as sole source of carbon by *Cb*. *Clostridium* species that utilize glycerol as a sole carbon source (e.g., *Cp* and *C. butyricum*) typically possess the 1,3-propanediol and/or 1,2-propanediol biosynthesis pathway that allows for efficient disposal of excess NAD(P)H generated from glycerol oxidation (Fig. 1). Perhaps, expressing the two-step 1,3-propanediol pathway alongside Gldh and DhaK in *Cb* might confer on *Cb*, the ability to metabolize glycerol as a sole carbon source. Given the current crude glycerol glut as a result of the rapidly expanding biodiesel industry and also the generation of microbial inhibitory compounds during lignocellulosic biomass pretreatment, the findings reported here represent a significant progress towards engineering *Cb* strains capable of efficient glycerol metabolism and detoxification of lignocellulose-derived inhibitor, with concomitant production of butanol.

## Materials and methods

### Microorganisms and culture conditions

*Clostridium beijerinckii* NCIMB 8052 (*Cb*; ATCC 51743) and *Clostridium pasteurianum* (*Cp*; ATCC 6013) were obtained from the American Type Culture Collection (Manassas, VA). Laboratory stocks of these microorganisms were maintained as spore suspensions in sterile, double-distilled water at 4 °C. *Cb* and *Cp* spores were revived and propagated as previously described [30]. Spores (200 µL) were heat-shocked at 75 °C for 10 min, cooled on ice for 2 min, and then inoculated into 10-mL anoxic tryptone–glucose–yeast extract (TGY) medium. *Cb* and *Cp* cultures were incubated at 35 °C in an anaerobic chamber (Coy Laboratory Products, Inc., Ann Arbor, MI) with a modified atmosphere of 82% N_2_, 15% CO_2_, and 3% H_2_ until optical density (OD_600_) reached ~ 0.9–1.1 [30]. Actively growing cultures (10%, v/v) were subsequently transferred to fresh TGY medium and incubated for 3–4 h (until cultures reached an OD_600_ ~ 1.1) to increase the pre-culture volume.

*Escherichia coli* DH5α (*Ec*) from New England Biolabs (Ipswich, MA) was used to maintain recombinant plasmids*. Ec* DH5α strains carrying recombinant plasmids were grown aerobically by incubation in shaken flasks at 25 °C and 144 rpm for 16 h in Lysogeny broth (LB) containing ampicillin (50 μg/mL).

### Generation of recombinant glycerol dehydrogenase (*dhaD1* and *gldA1*) and dihydroxyacetone kinase (*dhaK*) gene constructs

Gly_5_ peptide linker was used to covalently tether the *Cp* genes. Protein secondary structure prediction was performed using an online software—Protein Homology/analogy Recognition Engine (Phyre^2^ version 2.0; Structural Bioinformatics Group, Imperial College, London) to determine which terminus to include the Gly_5_ peptide linker. Inclusion of Gly_5_ peptide linker at the appropriate end allows sufficient gap between adjacent proteins and minimizes protein misfolding following expression of recombinant proteins.

We performed protein alignment using the NBCI Blast*p* algorithm and Visual Molecular Dynamics software (VMD version 1.9.1; Theoretical and Computational Biophysics Group, University of Illinois at Urbana-Champaign) to compare primary and secondary structures, respectively, among *Cp* Gldhs, and between *Cp* and *Cb* DhaK (Additional file 1: Table S2). The VMD software generates a root mean square deviation (RMSD) value that predicts the similarity between secondary structures of two proteins (Additional file 1: Tables S1, S2, S3). Proteins with closely matched secondary structures, when aligned, are expected to have lower RMSD value (e.g., *dhaD1* and *gldA1* in Additional file 1: Table S2).

**Fig. 9:**
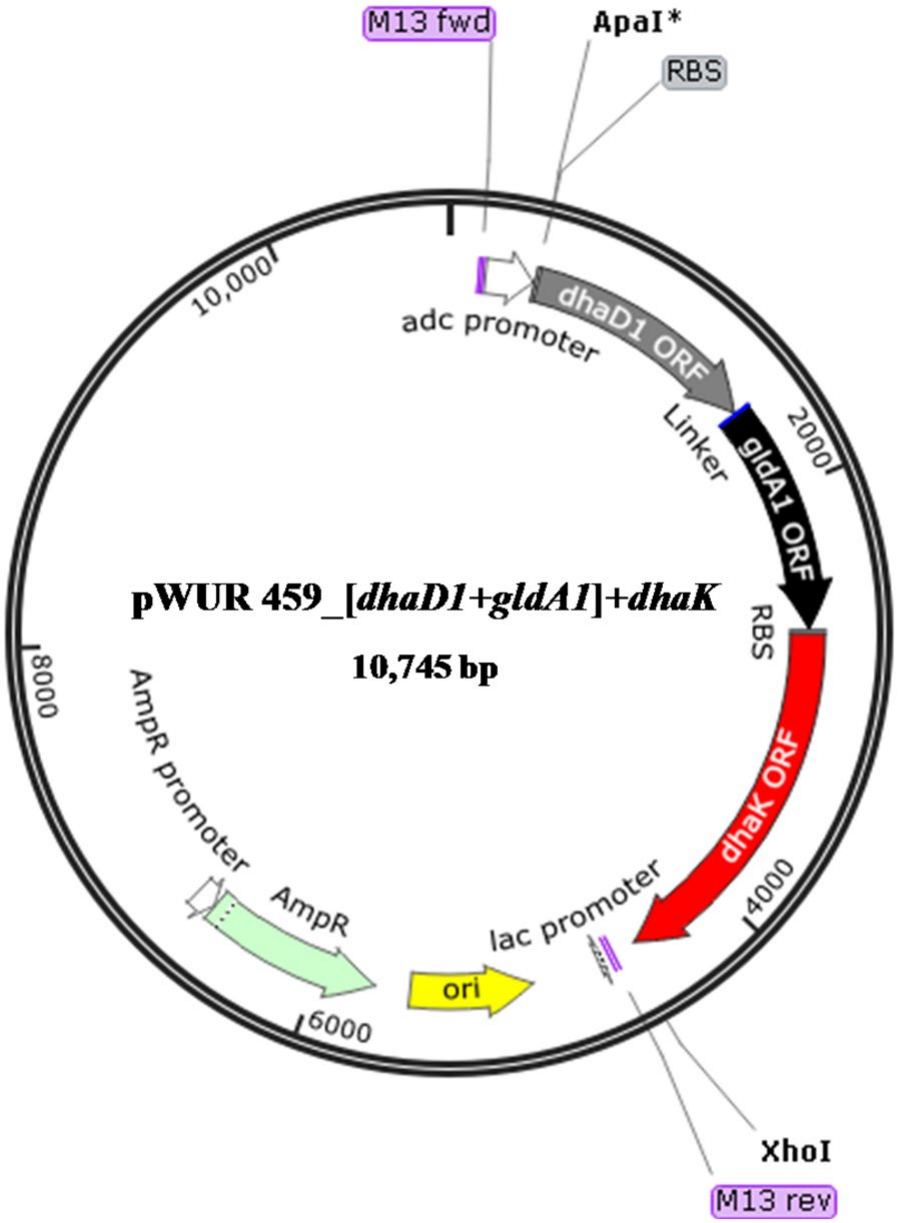
Schematic representation of recombinant plasmid bearing the construct *Cb* pWUR459_[(*dhaD1 *+ *gldA1*) *dhaK*]. All constructs were generated based on the same plan. The recombinant Cb strains bearing constitutively expressed constructs were based on the plasmid pWUR460, which bears the constitutive thiolase promoter, whereas pWUR459 bears inducible *adc* promoter

The DNA sequences for *Cp dhaD1, gldA1,* and *dhaK* were obtained from the EMBL-European Bioinformatics Institute and then cloned systematically into *Cb* expression plasmids. Nested PCR and splicing by overlap extension (SOE) PCR were used to amplify each gene from *Cp* gDNA to generate the fused gene constructs. Primers were designed to introduce a Gly_5_ peptide linker and a ribosome binding site, and to facilitate cloning of the fused gene construct into the *Clostridium*-*Ec* shuttle plasmids pWUR459 and pWUR460 at both the 5′ ApaI or NcoI site and the 3′ EcoRI or XhoI site (Fig. 9). Primer sequences are listed in Additional file 1: Table S4. All PCR reactions were performed using PrimeSTAR GXL DNA polymerase (Clontech, Mountain View, CA) and an iCycler™ Thermal Cycler (Bio-Rad, Hercules, CA). Each 50-µL reaction contained 1X PrimeSTAR GXL buffer, 250 µM dNTPs, 0.5 µM primers, ~ 5 ng/µL DNA template, and 1.25 U PrimeSTAR GXL DNA Polymerase.

The cycling conditions for the nested PCR reactions used to amplify each gene from *Cp* gDNA were: (1) 98 °C for 2 min (initial denaturation), (2) five cycles of 98 °C for 20 s, Annealing temperature 1 (AT1—of the portion of primer that anneals to gDNA) for 20 s, 72 °C for 30 s, (3) 30 cycles of 98 °C for 20 s, annealing temperature 2 (AT2—of entire primer sequence) for 20 s, 72 °C for 30 s (denaturation, annealing, and extension); (4) 72 °C for 5 min (final extension); and (5) 4 °C for 10 min. The fused gene constructs were assembled using a modified two-step SOE-PCR [31]. In Step 1 SOE-PCR, gene 1 and gene 2 PCR amplicons (e.g., *dhaD1* and *dhaK*) from the nested PCR reactions served as both primers and templates (forward- and reverse- templating fragments, respectively), which generated the fused gene construct (i.e., [*dhaD1 *+ *dhaK*]). Cycling conditions for Step 1 SOE-PCR were: (1) 98 °C for 2 min; (2) 15 cycles of 98 °C for 1 min, annealing temperature of overlap region [nucleotide sequence in the region where the two genes were linked] for 2 min, 72 °C for 3 min; (3) 72 °C for 10 min; (4) 4 °C for 10 min. The forward primer for gene 1 (e.g., dhaD1-F) and the reverse primer for gene 2 (e.g., dhaK-R) were then added to the Step 1 SOE-PCR reaction, and the following cycling conditions were used for Step 2 SOE-PCR: (1) 98 °C for 2 min; (2) 30 cycles of 98 °C for 1 min, annealing temperature of primer pair for 2 min, 72 °C for 3 min; (3) 72 °C for 5 min; (4) 4 °C for 10 min.

Each insert (*dhaD1, gldA1, gldA1 *+ *dhaK, dhaD1 *+ *gldA1,* [(*dhaD1 *+ *gldA1*) *dhaK*]) was then spliced into the *Clostridium*-*Ec* shuttle plasmids, pWUR460 or pWUR459, to permit transcription by a constitutive thiolase promoter or inducible acetoacetate decarboxylase promoter (from *C. acetobutylicum*), respectively [25]. Representative schematics of recombinant plasmids are shown in Fig. 9 and Additional file 1: Figure S1.

In summary, *Cp dhaD1* and *gldA1*, which exhibit the closest secondary structure similarity among *Cp gldhs*, were tethered at their C-terminus and N-terminus, respectively, to generate a fused construct [*dhaD1 *+ *gldA1*]. The insert was then spliced into each of pWUR460 and pWUR459. Furthermore, *dhaD1* and *gldA1* were separately tethered to *dhaK* at their C-and N-termini, respectively, to generate [*dhaD1 *+ *dhaK*] and [*gldA1 *+ *dhaK*], which were then spliced into both pWUR460 and pWUR459. Further, the fused *gldh* construct [*dhaD1 *+ *gldA1*] was linked to *dhaK* to create [(*dhaD1 *+ *gldA1*) *dhaK*], which was then spliced into pWUR459 for inducible expression as a fused protein. Additionally, *dhaD1* and *gldA1* inserts were separately spliced into pWUR460 and pWUR459 to be expressed as single (unfused) Gldh proteins.

### Restriction digestion and ligation of PCR amplicons into pWUR459/460 plasmid vectors

Plasmids and amplicons were digested with *Apa*I and *Xho*I, respectively (New England Biolabs, Ipswich, MA). A 50-µL digestion reaction consisted of nuclease-free water, Cutsmart buffer (5 µL; New England Biolabs, Ipswich, MA), *Apa*I (1 µL), and DNA samples (0.02 µg/mL). After overnight incubation at 25 °C, 1 µL *Xho*I was added to the reaction and then incubated at 37 °C for 4 h. The digested vectors and PCR products were purified by agarose gel electrophoresis using the GenCatch advanced PCR extraction kit (Epoch Life Science, Sugar Land, TX). Following restriction digestion and purification, linear plasmids and amplicons were ligated to generate recombinant plasmids. A 10-µL ligation reaction (insert: vector ratio of 10:1) consisted of 10× T4 ligase buffer (1 µL), linearized plasmid and insert (0.02–0.5 pmol), T4 DNA ligase (0.5 µL; New England Biolabs, Ipswich, MA), and nuclease-free water. The ligation reaction was incubated overnight at 16 °C, and then heat-inactivated at 65 °C for 10 min, and chilled on ice.

### Transformation of *E. coli* DH5α and *Cb* with recombinant plasmids

Electro-transformation of *E. coli* DH5α was conducted according to standard protocols. Colonies were selected on LB agar plate supplemented with ampicillin (50 μg/mL). Following isolation of recombinant plasmids from *E. coli* DH5α, the presence of desired inserts was verified by PCR, restriction digestion analysis, and DNA sequencing (Eurofins genomics, Louisville, KY).

To prepare electro-competent *Cb* cells, spores suspension (200 µL) was heat-shocked as previously described [22, 30]. *Cb* culture was then grown according to the previously described protocol [30]. The culture was then plated onto semi-solid TGY agar [0.5% (w/v)] and incubated until single colonies appeared. A single colony was inoculated into 10-mL TGY broth and incubated for 10–12 h. After 12 h, the actively growing *Cb* culture (10%, v/v) was then transferred into a fresh TGY broth and incubated until OD_600_ reached 0.6–0.8. Cells were harvested by spinning at 4000×*g* and 4 °C for 6 min. Cell pellets were washed once with electroporation buffer (50 mL) containing 270 mM sucrose, 1 mM MgCl_2_, 5 mM KH_2_PO_4_, and 10% w/v PEG-8000. Electro-competent *Cb* cells were re-suspended in 2 mL of the electroporation buffer and incubated on ice for 5 min.

To transform *Cb*, 10 µg of recombinant plasmid was gently mixed with 400 µL of the freshly prepared electro-competent *Cb* in a pre-chilled 0.2-cm electroporation cuvette. Electroporation was performed at 2.5 kV, 25 µF capacitance, and infinite resistance in a Bio-Rad Gene Pulser Xcell™ electroporator [32]. After electroporation, cells were diluted in 4 mL of TGY medium and incubated anaerobically at 35 °C for 6 h. The resulting cells were pelleted by centrifugation at 3000×*g* for 5 min and then mixed with 30-mL semi-solid TGY agar containing 25 µg/mL erythromycin, poured into two separate petri dishes and incubated in an anaerobic chamber for 48–72 h. Subsequently, colonies were selected, transferred onto fresh semi-solid TGY agar containing 25 µg/mL erythromycin), and grown for another 12–24 h. Then, colonies from each transformant were transferred into culture tubes containing TGY broth supplemented with 25 µg/mL erythromycin. The presence of the desired inserts was verified by PCR, restriction digestion analysis, and DNA sequencing (Eurofins genomics, Louisville, KY).

To prepare routine laboratory stocks of *Cb* transformants, 6% (v/v) actively growing cells were inoculated into TGY broth containing 50 g/L glucose supplemented with erythromycin (25 µg/mL) supplementation, and then grown for 7–8 days to allow for complete sporulation of the cells. At the end of fermentation, spores were harvested by centrifugation at 3000×*g* and 4 °C for 15 min. The pellets were washed 5–8 times with sterile distilled water, and then suspended in sterile distilled water, and stored at 4 °C.

### Batch fermentation to evaluate butanol production, glycerol utilization, and the furfural tolerance of recombinant *Cb* strains

Spores (200 µL) of recombinant *Cb* strains were heat-shocked and inoculated into TGY broth supplemented with 25 µg/mL erythromycin as previously described [22, 30]. *Cb* pre-culture was prepared according to the method of Ezeji et al. [33] using actively growing *Cb* cultures grown in TGY broth containing 25 µg/mL erythromycin. Butanol fermentation was conducted in loosely capped 50-mL Pyrex culture bottles using 6% (v/v) of the pre-culture in 50 mL of modified P2 medium [33] supplemented with 20 µg/mL erythromycin. The carbon source in the P2 medium was modified as follows: (i) glucose + glycerol (1:2 molar ratio; 36 g/L glucose and 36.1 g/L glycerol, respectively), (ii) glucose-only (60 g/L), and (iii) glucose + glycerol [1:2 molar ratio as in (i) above, with furfural supplementation (2,3,4,5, and 6 g/L)]. For furfural-supplemented cultures (iii above), at 10–12 h (OD_600_ of 0.9), the cultures were pulse-fed with furfural to the stated final concentrations. All experiments were performed in triplicate. During the course of fermentation, sample aliquots (2 mL) were taken at every 6- to 12-h intervals for analysis of cell growth, culture pH, and the concentrations of butanol, acetone, ethanol, acetic and butyric acids, glucose and glycerol, furfural, and furfuryl alcohol (see analytical methods section for details).

### Glycerol dehydrogenase assay

The activity of recombinant Gldh was determined as previously described by measuring the reduction of a colorless tetrazolium salt to purple formazan [34, 35]. Recombinant cells growing at mid-exponential phase (16 h) and mid-stationary phase (36 and 48 h) were harvested and washed with 100 mM KHCO_3_ buffer. Cell pellets were lysed in a Qiagen Tissue Lyser LT (Qiagen, Hilden, Germany) at 50 oscillations/s for 3 min, with addition of 0.1-mm-diameter zirconia/silica beads (BioSpec, Bartlesville, OK). Cell debris was removed by centrifuging the mixture at 10,000×*g* and 4 °C for 5 min. A 300-µL reaction mixture consisting 100 mM KHCO_3_ (pH 9.5), 250 µg/mL gelatin, 100 mM glycerol, 1 mM NAD^+^, 1 mM 2-*p*-iodo-3-*p*-nitrophenyl-5-phenyl-2H-tetrazolium chloride (INT), and 0.065 mM phenazine methosulfate (PMS) was prepared. The reaction mixture and the quartz cuvette used for the assay were pre-incubated at 37 °C in a water bath and a DU^®^ 800 spectrophotometer (Beckman Coulter Inc., Brea, CA), respectively. To start the reaction, 100-µL crude cell extract was added to 300 µL of pre-warmed reaction mixture in a cuvette. Absorbance (580 nm) was measured every 1 min for 20 min and then subtracted from those of the control (blank) consisting of 100-µL sterile distilled water and 300-µL reaction mixture.

A graph of absorbance versus time was plotted and the initial velocity (mM INT formazan formed/min) was determined according to a previously described protocol, using the extinction coefficient of formazan (12,300/M^/^cm @ 580 nm; [36, 37] and pathlength (1 cm). Protein concentration was estimated according to the method described by Bradford [38] using a commercial reagent (Amresco, Solon, OH). Bovine serum albumin was used as a standard. One unit of specific enzyme activity was defined as µmol INT formazan formed per min per mg protein.

### Dihydroxyacetone kinase assay

The activity of DhaK was determined in a coupled assay with glycerol-3-phosphate dehydrogenase by monitoring the rate of ATP-dependent oxidation of NADH to NAD^+^ monitored at 340 nm [39]. In the coupled assay, DHAP (the product of ATP-dependent DhaK reaction) is reduced to glycerol-3-phosphate by NADH-dependent glycerol-3-phosphate dehydrogenase. *Cb* cells growing to mid-exponential and stationary phases in glycerol-supplemented P2 medium were harvested and washed with 10 mL of ice-cold 20 mM 2-(N-morpholino)ethanesulfonic acid buffer (MES; pH 6.5). The cell pellet was lysed in a Qiagen Tissue Lyser LT (Qiagen, Hilden, Germany) at 50 oscillations/s for 3 min. A 350-µL reaction mixture, which consisted of 5 µg/mL α-glycerophosphate dehydrogenase, 20 mM MgCl_2_, 10 mM 2,2-dipyridyl, 1 mM KCN, 0.1 M imidazole (pH 7.5), 2 mM MES (pH 6.5), 4 mM dihydroxyacetone, 10 mM ATP, and 0.1 mM NADH was prepared. The reaction mixture and quartz cuvettes used for the assay were pre-incubated at 30 °C for 5 min in a water bath and a DU^®^800 spectrophotometer (Beckman Coulter Inc., Brea, CA), respectively. To start the reaction, 50-µL crude *Cb* cell-free extract was added to 350 µL of pre-warmed reaction mixture. Absorbance (340 nm) was measured every 2 to 5 min for 40 min and then subtracted from those of the control blank (50-µL sterile distilled water and 350-µL reaction mixture).

The activity of DhaK was calculated based on the extinction coefficient of NADH (6220/M/cm @ 340 nm; [40]) and path length (1 cm). Protein concentration was estimated as described above (glycerol dehydrogenase assay). One unit of specific DhaK activity was defined as the µmol NADH oxidized per min per mg protein.

### Analytical methods

Cell growth was determined by measuring optical density at 600 nm (OD_600_) using a DU^®^ 800 spectrophotometer (Beckman Coulter Inc., Brea, CA). Changes in the concentrations of furfural and furfuryl alcohol were determined by monitoring changes in wavelength using a DU^®^ 800 spectrophotometer. The maximum absorption spectra of furfural and furfuryl alcohol are at 275 and 220 nm, respectively. Furfural and furfuryl alcohol concentrations were further validated by High Performance Liquid Chromatography (HPLC) using a Waters 2796 Bioseparation Module equipped with Photodiode Array Detector (PDA; Waters, Milford, MA) and a 3.5-μm Xbridge C18, 150 mm × 4.6 mm column (Waters, Milford, MA). Samples were eluted using a gradient mobile phase of acetic acid [0.3% (v/v) in HPLC-grade water] and HPLC-grade methanol, operated at a flow rate of 0.6 mL/min as previously described [10].

Glycerol concentration was quantified by HPLC using the Waters 2796 Bioseparations Module equipped with a photodiode array (PDA) detector (Waters, Milford, MA), and a Prevail™ Carbohydrates ES Column-W 250 mm × 4.6 mm × 5 µm column (Grace Davison Discovery Science, Deerfiled, IL) maintained at 30 °C in series with an All-Guard™ Cartridge System (Alltech Associates Inc., Deerfield, IL). The mobile phase was acetonitrile [78% (v/v)] and water [22% (v/v)] at a flow rate of 1 mL/min and isocratic pump mode. Sample temperature and injection volume were 25 °C and 10 µL, respectively. Glucose concentrations were quantified using the 3,5-dinitrosalicylic acid (DNS) method [41]. Determination of glucose concentration was performed using a Waters 2796 Bioseparation module equipped with Evaporative Light Scattering Detector (ELSD; Waters, Milford, MA) and a 9-μm Aminex HPX-87P, 300 mm × 7.8 mm column maintained at 65 °C in series with an Aminex deashing guard column (4.6 mm internal diameter × 3 cm long; Bio-Rad, Hercules, CA). The mobile phase was HPLC-grade water at a flow rate of 0.6 mL/min [13].

The concentrations of acetone, butanol, ethanol, acetic acid, and butyric acid, were determined using a 7890A Agilent gas chromatograph (Agilent Technologies Inc., Wilmington, DE) equipped with a flame ionization detector (FID) and 30 m (length) × 320 μm (internal diameter) × 0.50 μm (HP-INNOWax film) J × W 19091N-213 capillary column as described previously Agu et al. [10]. ABE yield was defined as total ABE produced (in grams) per grams of glucose (or glucose + glycerol) utilized. Productivity was calculated as the concentration (g/L) of ABE produced per hour.

### Statistical analysis

The General Linear Model of Minitab version 17 (Minitab Inc., State College, PA) was used for all statistical analyses to compare differences between treatments. Differences in growth, product yields and productivities, rate of furfural reduction, and the concentrations of sugars, furfural and furfuryl alcohol, acetone, butanol, ethanol, and ABE were compared. ANOVA was conducted at different fermentation time points. Fisher’s least significant difference at 90% confidence interval was applied to pair-wise comparison to separate means.

## Additional file

**Additional file 1: Table S1.** Similarities (%) between *Cp* and *Cb* Gldh protein sequences. NCBI Blast*p* algorithm was used for alignment. **Table S2.** Root mean square deviation (RMSD) values for secondary structure alignment of *Cp* Gldhs. **Table S3.** Comparison of DhaK protein sequences between *Cp* and *Cb* DhaK proteins. NCBI Blast*p* algorithm was used for alignment. **Table S4.** List of primers and PCR protocols used to generate constructs. **Figure S1.** Schematics of some recombinant plasmids that were used to transform electrocompetent *Cb*. **Figure S2.** Enzyme–substrate conduit demonstrating putative metabolic function of *Cp* glycerol catabolic pathway engineering in *Cb*. Engineering of two Gldh as fused protein was designed to increase the active site concentration per location, thereby improving the efficiency of NAD(P)H generation during glycerol catabolism. *DHA* dihydroxyacetone, *DHAP* DHA phosphate, *DhaK* DHA kinase, *DhaD1 and GldA1 Cp* Gldh. **Figure S3.** DNA electrophoresis gel image to confirm PCR amplicons. Lanes (1) *dhaD1*, (2) *gldA1*, (3) *dhaD1+gldA1*, (4) DNA ladder, (5) *dhaK*, and (6) [*dhaD1+gldA1*] + *dhaK.* **Figure S4.** Furfural detoxification profile of *C. berijerinckii*-pWUR460_*dhaD1+gldA1* during the fermentation of glucose + glycerol (1: 2 molar ratio) challenged with 3 g/L (A), 4 g/L (B), 5 g/L (C), and 6 g/L furfural (D). (FF: Furfural; FA: Furfuryl alcohol).
