# Supplementary material for: Metabolic engineering of Clostridium beijerinckii to improve glycerol metabolism and furfural tolerance
Source: Biotechnol Biofuels. 2019 Mar 9;12:50. doi: 10.1186/s13068-019-1388-9 (PMC6408787; doi:10.1186/s13068-019-1388-9)
Supplement: Supplementary file 1 — Additional file 1: Table S1. Similarities (%) between Cp and Cb Gldh protein sequences. NCBI Blastp algorithm was used for alignment. Table S2. Root mean square deviation (RMSD) values for secondary structure alignment of Cp Gldhs. Table S3. Comparison of DhaK protein sequences between Cp and Cb DhaK proteins. NCBI Blastp algorithm was used for alignment. Table S4. List of primers and PCR protocols used to generate constructs. Figure S1. Schematics of some recombinant plasmids that were used to transform electrocompetent Cb. Figure S2. Enzyme–substrate conduit demonstrating putative metabolic function of Cp glycerol catabolic pathway engineering in Cb. Engineering of two Gldh as fused protein was designed to increase the active site concentration per location, thereby improving the efficiency of NAD(P)H generation during glycerol catabolism. DHA dihydroxyacetone, DHAP DHA phosphate, DhaK DHA kinase, DhaD1 and GldA1 Cp Gldh. Figure S3. DNA electrophoresis gel image to confirm PCR amplicons. Lanes (1) dhaD1, (2) gldA1, (3) dhaD1+gldA1, (4) DNA ladder, (5) dhaK, and (6) [dhaD1+gldA1] + dhaK. Figure S4. Furfural detoxification profile of C. berijerinckii-pWUR460_dhaD1+gldA1 during the fermentation of glucose + glycerol (1: 2 molar ratio) challenged with 3 g/L (A), 4 g/L (B), 5 g/L (C), and 6 g/L furfural (D). (FF: Furfural; FA: Furfuryl alcohol). [file 13068_2019_1388_MOESM1_ESM.doc]

**Metabolic engineering of *Clostridium beijerinckii* to improve glycerol metabolism and furfural tolerance**

Chidozie Victor Agu^1,2^, Victor Ujor^3^, and Thaddeus Chukwuemeka Ezeji^1*^

**SUPPLEMENTARY MATERIALS**

**Table S1:** Similarities (%) between *Cp* and *Cb* Gldh protein sequences. NCBI Blast*p* algorithm was used for alignment.

| **Protein sequence identity (%)** | ***Cb* GldA** | ***Cp* DhaD1** | ***Cp* DhaD2** | ***Cp* GldA1** | ***Cp* GldA2** |
| --- | --- | --- | --- | --- | --- |
| *Cp* DhaD1 | 51 | 100 | 43 | 29 | 27 |
| *Cp* DhaD2 | 54 | 43 | 100 | 28 | 36 |
| *Cp* GldA1 | 27 | 29 | 28 | 100 | 27 |
| *Cp* GldA2 | 37 | 27 | 36 | 27 | 100 |

**Table S2:** Root mean square deviation (RMSD) values for secondary structure alignment of *Cp* Gldhs.

| **RMSD** | ***Cp* DhaD1** | ***Cp* GldA1** | ***Cp* DhaD2** | ***Cp* GldA2** |
| --- | --- | --- | --- | --- |
| *Cp* DhaD1 | 0 | 3.53 | 5.81 | 98.49 |
| *Cp* GldA1 | 3.53 | 0 | 5.16 | 97.98 |
| *Cp* DhaD2 | 5.81 | 5.164 | 0 | 98.83 |
| *Cp* GldA2 | 98.49 | 97.98 | 98.83 | 0 |

**Table S3:** Comparison of DhaK protein sequences between *Cp* and *Cb* DhaK proteins. NCBI Blast*p* algorithm was used for alignment.

| **Identity (%)** | **%Similarity with *Cp* DhaK** |
| --- | --- |
| *Cb* DhaK subunit *Cbei*_2148 | 55 |
| *Cb* DhaK L subunit *Cbei*_2149 | 35 |
| *Cb* DhaK phosphotransferase subunit *Cbei*_2150 | 0 |

**Table S4:** List of primers and PCR protocols used to generate constructs.

| **S/N** | **Insert** | **Primers (5**' **to 3**'**) and PCR strategy** |
| --- | --- | --- |
| 1 | *dhaD1* | 12753-F: ATAGGGCCC**AGGAGG**TATCCATGGATGAGAAAAGCATTTATTTGTCC  dhaD1-R1: TAAAAAAATAAGAGTTACCATTTATTATTTACACATCCTCTTCTTTTCC  6017-R2: CGACCTCGAGAATTCACTATGAAACAATATTAAAAAAATAAGAGTTACCATTTATTAGA  **PCR strategy:** (1) *dhaD1* was amplified from *Cp* gDNA using primers 12753-F and DhaD1-R1 (AT1 = 54˚C, AT2 = 64˚C), (2) Next, amplicon (1) was re-amplified using 12753-F and 6017-R2 (AT1= 52˚C, AT2 = 67˚C). *NB: In all cases, including below, primers R1 & R2 were used to add terminator sequence to inserts* |
| 2 | *gldA1* | gldA1-F: ATAGGGCCCAGGAGGTATCCATGGATGAGTTATAGTGTTTTTTTACCAAG  6017-R1: TAAAAAAATAAGAGTTACCATTTATTAGACAGCTTTCACAGGC  6017-R2: CGACCTCGAGAATTCACTATGAAACAATATTAAAAAAATAAGAGTTACCATTTATTAGA  **PCR strategy:** (1) *gldA1* was amplified from *Cp* gDNA using primers gldA1-F and 6017-R1 (AT1 = 54˚C, AT2 = 66˚C), (2) Next, the amplicon from 1 was re-amplified above using gldA1-F and 6017-R2 (AT1 = 52˚C, AT2 = 67˚C) |
| 3 | *dhaD1 + gldA1* | 12753-F: ATAGGGCCC**AGGAGG**TATCCATGGATGAGAAAAGCATTTATTTGTCC  12753-R: ACCACCACCACCACC TTTACACATCCTCTTCTTTTCC  6017-F: AAA GGTGGTGGTGGTGGT ATGAGTTATAGTGTTTTTTTACCAAG  6017-R1: TAAAAAAATAAGAGTTACCATTTATTAGACAGCTTTCACAGGC  6017-R2: CGACCTCGAGAATTCACTATGAAACAATATTAAAAAAATAAGAGTTACCATTTATTAGA  **PCR strategy:** (1) *dhaD1* was amplified with primer pair 12753-F & R (AT1 = 54˚C, AT2 = 71˚C) (2) *gldA1* was amplified with primer pair 6017-F & R1 (AT1 = 54˚C, AT2 = 66˚C), then re-amplify the resulting amplicon with 6017-F & R2 (AT1 = 52˚C, AT2 = 67˚C). (3) *dhaD1* and *gldA1* amplicons were spliced via two step SOE PCR – Step 1: dhaD1 and gldA1 served as both primers and template (forward and reverse templating fragments, respectively). Annealing temp. = overlap region; step 2: amplicon resulting from step 1 SOE was used as template for normal PCR using primers 12753-F and 6017-R2 (AT = 67˚C) |
| 4 | [*dhaD1+gldA1*] + *dhaK* | 12753-F: ATAGGGCCC**AGGAGG**TATCCATGGATGAGAAAAGCATTTATTTGTCC  Gldh-R: cttgcaaCCTCCTacgctaTTAGACAGCTTTCACAGGCTC  dhaK-F1: GGAGGttgcaagat ATGAAAAAGATAATAAATAAACCAG  dhaK-F2: AAAGCTGTCTAAtagcgtA GGAGGttgcaagatATGAAAAAG  dhaK-R1: TAAAAAAATAAGAGTTACCATTTA CTACTTTATAACCTCTGAAATC  dhaK-R2: cgacctcgagaattcACTATGAAACAATAT TAAAAAAATAAGAGTTACCATTTACTACT  **PCR strategy:** (1) *dhaD1+gldA1* was re-amplified with primers 12753-F and Gldh-R (AT1 = 54˚C, AT2 = 72˚C), (2) *dhaK* was amplified from *Cp* gDNA with primers dhaK-F1 and dhaK-R2 (AT1 = 50˚C, AT2 = 62˚C), (3) Re-amplified PCR product from 2 using primers dhaK-F2 and dhaK-R2 (AT1 = 54˚C, AT2 = 67˚C), (4) PCR products 1 and 3 above were spliced via two step SOE-PCR; Step 1: AT of overlap region (69˚C) using PCR amplicons 1 and 3 as forward and reverse templating fragments; Step 2: the resulting amplicon from step 1 SOE was used as template for normal PCR with primers 12753-F and dhaK-R2 (AT = 67˚C).  *NB: AT of overlap region must be at least 65˚C for efficient SOEing* |
| 5 | *dhaD1 + dhaK* | 12753-F: ATAGGGCCC**AGGAGG**TATCCATGGATGAGAAAAGCATTTATTTGTCC  12753-R: ACCACCACCACCACC TTTACACATCCTCTTCTTTTCC  dhaK-F4: AAAGGTGGTGGTGGTGGT ATGAAAAAGATAATAAATAAACCAG  dhaK-R1: TAAAAAAATAAGAGTTACCATTTA CTACTTTATAACCTCTGAAATC  dhaK-R2: cgacctcgagaattcACTATGAAACAATAT TAAAAAAATAAGAGTTACCATTTACTACT  **PCR strategy:** (1) *dhaD1* was amplified from *Cp* gDNA using primers 12753-F and 12753-R (AT1 = 54˚C, AT2 = 71˚C), (2) *dhaK* was amplified using primers dhaK-F4 and dhaK-R1 (AT1 = 50˚C, AT2 = 62˚C); the resulting amplicon was re-amplified using dhaK-F4 and dhaK-F2 (AT1 = 50˚C, AT2 = 67˚C), (3) Spliced amplicons 1 and 2 by SOE PCR. Step 1: AT = 59˚C; Step 2: AT = 67˚C |
| 6 | *gldA1 + dhaK* | gldA1-F: ATAGGGCCCAGGAGGTATCCATGG ATGAGTTATAGTGTTTTTTTACCAAG  gldA1-R: ACCACCACCACCACC GACAGCTTTCACAGGCTCATTAC  dhaK-F5: GTCGGTGGTGGTGGTGGT ATGAAAAAGATAATAAATAAACCAG  dhaK-R1: TAAAAAAATAAGAGTTACCATTTA CTACTTTATAACCTCTGAAATC  dhaK-R2: cgacctcgagaattcACTATGAAACAATAT TAAAAAAATAAGAGTTACCATTTACTACT  **PCR strategy:** (1) *gldA1* was amplified from *Cp* gDNA using primer set gldA1-F and gldA1-R (AT1 = 54˚C, AT2 = 71˚C), (2) Amplified *dhaK* gene from *Cp* gDNA using primer set dhaK-F5 and dhaK-R1 (AT1 = 50˚C, AT2 = 62˚C), then re-amplified the resulting amplicon using primers dhaK-F5 and dhaK-R2 (AT1 = 50˚C, AT2 = 67˚C). (3) Spliced amplicons 1 and 2 by SOE PCR. Step 1: AT = 63˚C; Step 2: AT = 67˚C |
| 7 | *gldA2 + dhaD2* | 14565-F: TATGGGCCC**AGGAGG**ATATTACCATGGATGATAAAAATTAAGGCACCAG  14565-R: ACCACCACCACCACC TACTATAGCTTTCAACTTTAAACTACTAC  17742-F: GTAGGTGGTGGTGGTGGT ATGGTAAAATCAATTTACTCAC  17742-F1: CAATATTAAAAAAATAAGAGTTACCATTTA CTATTTAAATTGCTTTCCAAGTTC  17742-R2: GCGG CTCGAGGATCC ACTATGAAA CAATATTAAAAAAATAAGAGTTACC  **PCR strategy:** (1) *gldA2* was amplified from *Cp* gDNA using primer pair 14565-F & R (AT1 = 53˚C, AT2 = 69˚C), (2) Amplified gene dhaD2 with primer pair 17742-F & R1 (AT1 = 50˚C, AT2 = 64˚C), then re-amplify the resulting amplicon with 17742-F & R2 (AT1 = 49˚C, AT2 = 69˚C). (3) Spliced *gldA2* and *dhaD2* amplicons from 1 and 2 via two step SOE PCR – Step 1: *gldA2* and *dhaD2* amplicons served as both primers and template (forward and reverse templating fragments, respectively). Annealing temp. = overlap region; step 2 SOE: amplicon resulting from step 1 SOE was used as template for normal PCR using primers 14565-F and 17742-R2 (AT = 69˚C) |

**Fig. S1:** Schematics of some recombinant plasmids that were used to transform electrocompetent *Cb*.

**Fig. S2:** Enzyme-substrate conduit demonstrating putative metabolic function of *Cp* glycerol catabolic pathway engineering in *Cb*. Engineering of two Gldh as fused protein was designed to increase the active site concentration per location, thereby improving the efficiency of NAD(P)H generation during glycerol catabolism.. DHA = dihydroxyacetone; DHAP = DHA phosphate; DhaK = DHA kinase; DhaD1 and GldA1 = *Cp* Gldh.

**Fig. S3:** DNA electrophoresis gel image to confirm PCR amplicons. Lanes (1) *dhaD1*, (2) *gldA1*, (3) *dhaD1+gldA1*, (4) DNA ladder, (5) *dhaK*, and (6) [*dhaD1+gldA1*] + *dhaK.*

**Fig. S4:** Furfural detoxification profile of *C. berijerinckii*-pWUR460_*dhaD1+gldA1* during the fermentation of glucose + glycerol (1: 2 molar ratio) challenged with 3 g/L (A), 4 g/L (B), 5 g/L (C), and 6 g/L furfural (D). (FF: Furfural; FA: Furfuryl alcohol).
